# Effect of different adhesive strategies on the microtensile bond strength of dentin to indirect resin-based composite

**DOI:** 10.4317/jced.57094

**Published:** 2020-11-01

**Authors:** Guilherme Pinto, Lúcia Prieto, Josué-Junior Pierote, Laura Ferraz, João-Victor Câmara, Flávio-Henrique Aguiar

**Affiliations:** 1 Graduate student, Department of Restorative Dentistry, Piracicaba Dental School, State University of Campinas, Piracicaba, São Paulo, Brazil; 2 Postdoctoral student, Department of Restorative Dentistry, Piracicaba Dental School, State University of Campinas, Piracicaba, São Paulo, Brazil; 3 Master student, Department of Biological Sciences, Bauru School of Dentistry, University of São Paulo, Bauru, São Paulo, Brazil; 4 Titular Professor, Department of Restorative Dentistry, Piracicaba Dental School, State University of Campinas, Piracicaba, São Paulo, Brazil

## Abstract

**Background:**

To evaluate the microtensile bond strength of indirect resin composite bonded to dentin using five different adhesives strategies.

**Material and Methods:**

Forty specimens (Solidex) were produced and randomly into five groups with different adhesives strategies: (G1)- Single Bond Universal + etch + silane + RelyX Ultimate, (G2)- Single Bond Universal + silane + RelyX Ultimate, (G3)- Single Bond Universal + etch + RelyX Ultimate, (G4)- Single Bond Universal + RelyX Ultimate, and (G5)-Scotchbond Multi-purpose + RelyX ARC. After cementation the specimens were stored in 100% humidity for 24hours at 37°C. The specimens were sectioned perpendicular to the adhesive interface to obtain beams and submitted to microtensile test. Microtensile values were expressed in MPa and analyzed by one-way ANOVA and multiple comparison Tukey tests (α=0.05).

**Results:**

The mean bond strength in MPa groups were: G1=11,48, G2=14,15, G3=16,95, G4=17,03 and G5=16,80. Statistical analysis showed that the bond strength values were not significantly affected by the different adhesive strategies.

**Conclusions:**

Cementation of dentin to indirect resin composite cannot be significantly affected by different adhesive strategies used. The specimens treated with silane, etch associated with Single bond universal did not increase bond strength values.

** Key words:**Adhesives, dental cements, dentin.

## Introduction

Since the first methacrylate-based were developed changes are more focused on the polymeric matrix of these materials, to develop systems with reduced polymerization shrinkage (1). The use of indirect resin composites restorations ensures a better bond strength because the impact of polymerization shrinkage on adhesion in dentin is insignificant (2). The additional polymerization process enhances the physical and mechanical properties of indirect composite resins (3), besides to decrease a chemical bonding capacity because the quantity of residual free carbon bonds decreases.

To improve bond strength of indirect composite resins to dentin different surface treatments have been proposed. Silane is a bifunctional molecule that can react with the methacrylate groups of the adhesives resins (4). The applications of silane increase the surface energy of ceramic and the wettability of the resin materials, achieving bonding both physically and chemically (5).

The success of indirect composite resin restorations also depends on the pretreatment of the tooth surface. The dentin surface treated with phosphoric acid undergoes chemical and physical alterations that allow for chemical and micromechanical bonding with the adhesive materials (6). The efficiently of the bonding procedures occurs when resin monomers impregnated into partially demineralized dentin create a dentin-resin zone (7).

Several of dental adhesives know as “universal” adhesives systems are being marketed, these materials can be applied either with the etch-and-rise or the self-etch procedures (8). These universal adhesives incorporate the monomers that are able of producing chemical bonding to the dental substrates (9).

The literatures are still scarce with regard to the longevity of bonds produced by indirect resin composites restorations and which adhesive strategies influence the final bond strength of these indirect restorations.

Thus, the aim of this study was designed to evaluate the microtensile bond strength of indirect resin composite bonded to dentin using five different adhesives strategies. The following null hypothesis was tested: The bond strength results are not influenced by the adhesives strategy selected.

## Material and Methods

Forty bovine incisors were selected for the study. The teeth were cleaned any residual soft tissue and debris was removed using a scaler. The teeth were then rinsed and stored in distilled water with 0.1% thymol solution at 4°C. The root portions were removed using a low-speed diamond saw. Vestibular surface was then ground with wet 600,800,1000 and 1200- grit silicon carbide abrasive papers (Buehler, Lake Bluff, IL, USA) to achieve flat surface in dentin (25 mm2 of area). After the cleaned the teeth were stored in an aqueous solution until start of the experiment.

The block specimens were obtained by placing an indirect resin-based composite Solidex (Shofu Dental Corporation, Quioto, Japan) inside silicon molds Express XT (3M ESPE, Sumaré, São Paulo, Brazil) 5mm deep and 5mm in diameter. The specimens were made in 2mm thick increments and light cured for 20 seconds (Radii-cal, SDI Dental Product SDI, Bayswater, Vitoria, Australia, with a 1200 mW/cm2 output). The specimens were then removed from the mold and subjected to an additional cycle of polymerization in an oven (FDG- Lux) for 3 minutes. All specimens surface was abraded to an air-bone particle abrasion with 50 µm Al2O3 the device was kept 20 cm away from the specimen’s surface for 5 seconds. The specimens were then cleaned in an ultrasonic bath to remove all debris.

The teeth were randomly divided into five groups (n=8/per group):

G1: (Single Bond Universal + Etch + Silane+ RelyX Ultimate): The indirect composite blocks were treated with RelyX Ceramic Primer (3M ESPE, Sumaré, São Paulo, Brazil) for 60 seconds and dried with air spray for 60 seconds. The dentin surface was etched for 15 seconds with 37% phosphoric acid gel, and then washed with a water/air spray for 15 seconds. The excess water was dried from the dentin with a wet cotton pellet. The Single bond Universal adhesive systems (3M ESPE, Sumaré, São Paulo, Brazil) were applied in dentin and dried with air spray for 5 seconds. The RelyX Ultimate resin cement (3M ESPE, Sumaré, São Paulo, Brazil) were mixed according to manufacturers instructions and placed on the indirect composite blocks. The blocks were then luted on the respective bonded dentin surfaces. The excess cement was removed from the margins and light polymerized for 15 seconds from each side.

G2: (Single Bond Universal + Silane + RelyX Ultimate): The indirect composite blocks were treated with RelyX Ceramic primer for 60 seconds and dried with air spray for 60 seconds. The Single bond Universal adhesive systems were applied in dentin and dried with air spray for 5 seconds. The RelyX Ultimate resin cement were mixed according to manufacturers instructions and placed on the indirect composite blocks. The blocks were then luted on the respective bonded dentin surfaces. The excess cement was removed from the margins and light polymerized for 15 seconds from each side.

G3: (Single Bond Universal + Etch + RelyX Ultimate): The dentin surface was etched for 15 seconds with 37% phosphoric acid gel, and then washed with a water/air spray for 15 seconds. The excess water was dried from the dentin with a wet cotton pellet. The Single bond Universal adhesive systems were applied in dentin and dried with air spray for 5 seconds. The RelyX Ultimate resin cement were mixed according to manufacturers instructions and placed on the indirect composite blocks. The blocks were then luted on the respective bonded dentin surfaces. The excess cement was removed from the margins and light polymerized for 15 seconds from each side.

G4: (Single Bond Universal + RelyX Ultimate): The Single Bond Universal adhesive systems was applied in dentin and dried with air spray for 5 seconds. The RelyX Ultimate resin cement were mixed according to manufacturers instructions and placed on the indirect composite blocks. The blocks were then luted on the respective bonded dentin surfaces. The excess cement was removed from the margins and light polymerized for 15 seconds from each side.

G5: (Adper Scotchbond Multipurpose Plus + RelyX ARC) The indirect composite blocks were treated with RelyX Ceramic primer for 60 seconds and dried with air spray for 60 seconds. The dentin surface was etched for 15 seconds with 37% phosphoric acid gel, and then washed with a water/air spray for 15 seconds. The excess water was dried from the dentin with a wet cotton pellet. The activator, primer and catalyst of Adper Scotchbond Multipurpose Plus were applied to the dentin according to the manufacturer’s instructions. The RelyX ARC resin cement was mixed according to manufacturers instructions and placed on the indirect composite blocks. The blocks were then luted on the respective bonded dentin surfaces. The excess cement was removed from the margins and light polymerized for 15 seconds from each side.

After cementation procedures the specimens were bonded with cyanoacrylate glue (Super Bonder Gel, Loctite Ltd, São Paulo, Brazil) to a plastic base that was attached to a cutting machine (IsoMet 1000, Buehler Inc., Lake Bluff, IL, EUA). The specimens were positioned perpendicular to the disk. The specimens were sectioned in y and x direction using a slow-speed diamond disk. The beam specimens with a cross-sectional area of approximately 1mm2 were obtained. The dimensions of the beams were determined with a digital caliper (Mitutoyo Corp).

The beams were fixed with cyanoacrylate glue (Super Bonder Gel, Loctite Ltd, São Paulo, Brazil) to the fixtures of a universal testing machine (EZ-Test L, Shimadzu Co, Kyoto, Japan) and tested in tension at 0.5mm/min until fracture. After failure, the specimens were removed from a universal machine, measured with a digital caliper. The microtensile bond strength values were calculated in megapascals (MPa).

A one-way ANOVA was performed to analyze the effect of different adhesives strategies on the microtensile bond strength of luted indirect resin-based composite. Multiple comparisons were evaluated using Tukey tests. Statistical significance was set in advance at α=0.05.

## Results

The means and standard derivations of the microtensile bond strength values for each group are summarized in Table 1. The one-way ANOVA showed that the different adhesives strategies did not affected the bond strength for all the experimental groups. No failures occurred before testing for any of the experimental groups evaluated.

**Table 1:**
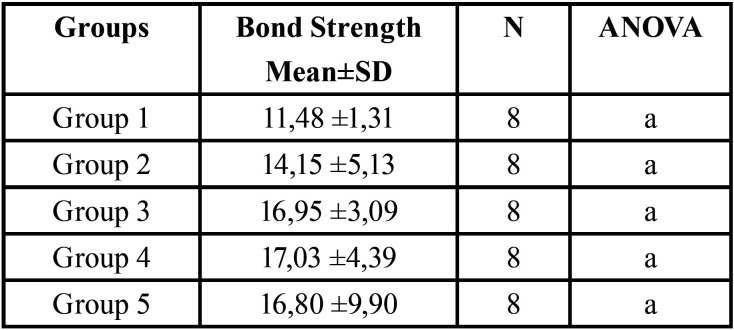
Mean Microtensile Bond Strength values (MPa) and Standard Deviations (SD) for indirect composite resins.

## Discussion

The adhesive and dentin set, which characterizes the hybrid layer, as well as the prosthesis and cement, were the main focus of this research aiming at the optimization of the bond strength of these interfaces, since the weaker one defines the final bond strength of the restoration, taking role in union stability.

An indirect resin-based composed of 53% of inorganic ceramic microfiller and 25% of co-polymers with multifunctional resin and 22% of conventional resin (Solidex) was associated with a self-etching adhesive (Single Bond Universal). To improve bond strength of indirect composite resins to dentin, different surface treatments have been proposed as the acid etching of dentin with 37% phosphoric acid gel and the indirect restoration silanization (RelyX Ceramic primer). The control group was treated with 37% phosphoric acid followed by Adper Scotchbond Multipurpose Plus and RelyX ARC resin cement. Microtensile bond strength (µTBS) was evaluated and the results showed that the adhesive strategy did not significantly affect the adhesion strength results of indirect resin-based composite to dentin, accepting the null hypothesis.

Dental adhesive technology has evolved in the past decades toward complex formulations with simplified clinical procedures. One of the latest innovations was the universal adhesives, which are presented in a single bottle which can be applied in etched or unetched enamel and dentin, as demineralization and priming occur simultaneously (10). The adhesion mechanism of the self-etch adhesives occurs through two bond mechanisms: micro-mechanical interlocking and chemical bonding. The micro-mechanical bonding contributes to provide strength against mechanical stress. The chemical interaction reduces hydrolytic degradation, keeping the marginal sealing of restorations for a longer period. This type of adhesion appears to be advantageous in terms of the durability of the restoration (11).

It has been proposed that the exposed collagen fibrils and opening up of the dentin tubules, after phosphoric acid etching, play an important role in creating the hybrid layer and effectively removing smear plugs to enable the formation of resin tags (12). However, acid etching does not always play a fundamental role in bond strength increase. In this study, no statistically significant differences were found when acid etching of dentin with 37% phosphoric acid was done or not prior to the application of self-etching adhesive. These results are in agreement with previous study that demonstrated that the performance of dentin acid conditioning does not interfere with the adhesive effectiveness of self-etching adhesives (13). This can be explained by the fact that the length of resin tags does not influence the bond strength of systems self-etching adhesives, since the resin monomers present in these products penetrate the collagen fibril network, forming a satisfactory bond (14).

Moreover, the efficiency of dentin etching depends more on the chemical composition of the adhesive than the effect of the phosphoric acid itself. In Single Bond Universal, methacrylate monomers (UDMA and GDMA) are replaced by phosphorised methacrylate monomers (MHP or MDP) to lower pH and have a self-etching property. MDP molecule has a long linear alkyl chain and phosphoric acid ester group. MDP is able to interact chemically with hydroxyapatite intensively and stably (15) This monomer forms a sTable nanolayer together with a deposition of sTable MDP-Ca salts at the adhesive interface (16), which increases its mechanical strength (16). In addition, MDP has shown not only chemical bonding to hydroxyapatite, but also to self-assemble into nanolayers, which has strong hydrophobic properties that protect the hybrid layer against hydrolytic degradation (15). Studies showed that MDP allows for a sTable chemical bond to dentin over the course of time, both *in vitro* (17) and *in vivo* (18,19). In addition, the Bond Universal single has polyalkenoic acid copolymer (PAC)in its composition in the percentage of up to 5%. PAC is capable of chemically bonding to hydroxyapatite in glass ionomer materials (20). A study showed that a PAC-containing patch had greater adhesion strength than a PAC-free adhesive with the same composition (21). Also, it seems that the association between PAC and MDP increases bonding ability (8).

Regarding the interface between cement and indirect composite, we can observe that several methods have been used in order to make the surface of indirect composite restorations more favorable to adhesion with the resin cement. Some of these techniques include airborne particles abrasion and conditioning with hydrofluoric (3) and signalization (22).

The most common method used in everyday clinical practice is silanization of indirect composite surface. Silanes are bifunctional molecules that are used to create a chemical bond between the methacrylate monomers of the resin cement matrix and the inorganic fillers of the indirect composite through the silanol group that reacts with silica on the glass filler surface, and the mathacrylate group in the silano molecule forms a covalent bond with the resinous matrix (3). In addition, the silane agent also renders the hydrophobic surface that results in increased wettability of the composite (23). After silanization, the application of an unfilled resin agent would improve the wettability of the indirect composite and allow the composite to flow into the irregularities of the precured composite.

Studies have shown that silanization results in a positive effect on the bond strength in the cementation of resin composites (24). However, no significant statistical differences were found in the present study between silanization or not. According Fuentes *et al.* (22), self-adhesive resin cements do not require application of intermediary agent (silane alone o silane plus bonding agent) to microretentive Filtek Z250 overlays to improve the bonding capacity of dentin-indirect composite complex. In the present study, this result can also be explained by the airborne particles abrasion because composite surface treatments are important for adhesion of indirect composite restorations (25).

In the present study aluminum oxide blasting was not a variable but a part of the bonding procedures. The performance of such a step may have influenced the results found in this study since, independent of the surface treatment used, no significant statistical. D’Arcangelo & Vanini (25) showed that roughening the composite area of adhesion, sandblasting, or both sandblasting and silanizing can provide statistically significant additional resistance to tensile load. Also, they suggest that sandblasting treatment was the main factor responsible in improving the retentive properties of indirect composite restorations (25), differences were found.

## Conclusions

Within the limitations of this *in vitro* study, the following conclusions were drawn:

- Cementation of dentin to indirect resin composite cannot be significantly affected by different adhesive strategies used.

- The composite resin indirect treated with silane or the etching of dentin with 37% phosphoric acid gel associated with Single bond universal did not increase bond strength values.
